# Robotic partial nephrectomy is associated with a significantly decreased rate of postoperative pseudoaneurysm compared to open and laparoscopic partial nephrectomy

**DOI:** 10.1007/s11701-024-01999-3

**Published:** 2024-06-04

**Authors:** Husny Mahmud, Boris Haitovic, Dorit E. Zilberman, Barak Rosenzweig, Menachem Laufer, Orith Portnoy, Eddie Fridman, Zohar A. Dotan

**Affiliations:** 1 Department of Urology, Affiliated to the Faculty of Medicine, Sheba Medical Center, Tel Aviv University, Tel Hashomer, Tel Aviv, Israel; 2 Unit of Interventional Radiology, Affiliated to the Faculty of Medicine, Sheba Medical Center, Tel Aviv University, Tel Hashomer, Tel Aviv, Israel; 3 Diagnostic Imaging Department, Affiliated to the Faculty of Medicine, Sheba Medical Center, Tel Aviv University, Tel Hashomer, Tel Aviv, Israel; 4 Pathology Department, Affiliated to the Faculty of Medicine, Sheba Medical Center, Tel Aviv University, Tel Hashomer, Tel Aviv, Israel

**Keywords:** Angiography, Laparoscopic partial nephrectomy, Open partial nephrectomy, Postoperative, Pseudoaneurysm, Renal artery, Robotic partial nephrectomy, Selective embolization

## Abstract

While partial nephrectomy offers oncologic efficacy and preserves renal function for T1 renal tumors, renal artery pseudoaneurysm (RAP) remains a rare but potentially life-threatening complication. This study compared RAP incidence across robotic-assisted (RAPN), laparoscopic (LPN), and open (OPN) partial nephrectomies in a large tertiary oncological center. This retrospective study analyzed 785 patients undergoing partial nephrectomy between 2012 and 2022 (398 RAPN, 122 LPN, 265 OPN). Data included demographics, tumor size/location, surgical type, clinical presentation, treatment, and post-operative outcomes. The primary outcome was RAP incidence, with secondary outcomes including presentation, treatment efficacy, and renal function. Seventeen patients (2.1%) developed RAP, presenting with massive hematuria (100%), hemorrhagic shock (5.8%), and clot retention (23%). The median onset was 12 days postoperatively. RAP occurred in 4 (1%), 4 (3.3%), and 9 (3.4%) patients following RAPN, LPN, and OPN, respectively (*p* = 0.04). Only operative length and surgical approach were independently associated with RAP. Selective embolization achieved immediate bleeding control in 94%, with one patient requiring a second embolization. No additional surgery or nephrectomy was needed. Estimated GFR at one year was similar across both groups (*p* = 0.53). RAPN demonstrated a significantly lower RAP incidence compared to LPN and OPN (*p* = 0.04). Emergency angiographic embolization proved effective, with no long-term renal function impact. This retrospective study lacked randomization and long-term follow-up. Further research with larger datasets and longer follow-ups is warranted. This study suggests that robotic-assisted partial nephrectomy is associated with a significantly lower risk of RAP compared to traditional approaches. Emergency embolization effectively treats RAP without compromising long-term renal function.

## Introduction

The surgical approach for clinical T1 renal masses has shifted from radical nephrectomy to partial nephrectomy (PN) for preservation of renal function [1–4]. The original PN was an open approach that was oncologically and surgically effective and safe compared to radical nephrectomy [1–3], yielding a significantly decreased rate of renal insufficiency [1–5]. The introduction of minimally invasive PN (MIPN), laparoscopic and robotic-assisted PN (RAPN) caused a major shift toward minimal invasiveness aimed at decreasing postoperative complication rates, allowing short hospital stay, and lessening recovery time [1–6]. RAPN has now become the most prevalent surgical approach for renal masses treated by partial nephrectomy.

Hemorrhage is the most common complication of partial nephrectomy [10]. A renal artery pseudoaneurysm (RAP) is a unique and rare hemorrhagic complication of partial nephrectomies [11], characterized by late bleeding and usually presenting as hematuria, flank pain, and anemia that is treated effectively by selective arterial embolization [11]. The etiology of RAP is undetermined, but it is probably related to arteriole laceration during the suturing of the renal bed after tumor removal that creates the RAP after the closure of the renal defect [11–13].

The selection of technique for performing a PN as a predictor for the subsequent likelihood of a RAP is controversial. Most series did not demonstrate any difference in the incidence of RAP in association with either an open, laparoscopic, or robotic approach [11–15]. One large systematic review that included 5229 patients who underwent PN demonstrated a significantly higher rate of RAPs among those patients treated by an MIPN compared to open partial nephrectomy (OPN) [11]. In the present study, we review the incidence, management, and outcomes of RAPs in a large surgical series of a tertiary oncological center, and compare the results achieved with it to those of the various alternative surgical approaches (LPN and OPN).

## Patients and methods

### Patients

Between January 2012 and December 2022, we carried out PNs on 785 patients with renal tumors clinical T1-3, of whom 398 (50.7%) patients underwent RAPNs, 122 (15.5%) underwent LPNs, and 265 (33.8%) underwent OPNs. The analyzed clinical data and imaging studies included demographic data, tumor characteristics (side, size, location, RENAL nephrometry score), surgical characteristics (warm ischemia time, estimated blood loss, operative time), and postoperative and hospitalization data. Moreover, radiographic and angiographic findings of patients diagnosed as having a RAP were reviewed to document the size of the pseudoaneurysm, its location in the kidney, the efficacy of the selected embolization, and the surgical outcomes.

### Ethics

This study was conducted with the Declaration of Helsinki and the International Council for Harmonisation Guidelines for Good Clinical Practice. The protocol approval number for this study is SMC-23–4146.

### Surgical techniques and outcomes

The primary endpoint of the study was the presence of a postoperative RAP. We documented the efficacy of the selected therapeutic approach for the RAP, as well as the postoperative and post-therapy renal function. All of the study patients were treated by five high-volume surgeons with extensive experience performing nephron-sparing surgery. The choice of surgical approach was according to the discretion of the surgeon. Our surgical techniques were described in detail elsewhere [4]. The robotic surgeries used the DaVinci Si or Xi surgical system. The patient’s demographics, tumor and surgical variables, as well as postoperative complications and outcomes were identified from the nephrectomy databases of the Department of Urology. Preoperative renal complexity was determined according to the RENAL nephrometry system [6]. The clinical data of the RAP included the presence of hematuria, flank pain, and hemodynamic stability at presentation, the time from the emergency room presentation to angiographic therapy, and its outcomes.

### Statistical analysis

In the descriptive statistical analysis, continuous variables were presented as means ± standard deviations (SDs) for those adhering to a normal distribution, and as medians with interquartile ranges (IQRs) for variables deviating from normality. Categorical variables were expressed as frequencies and percentages. Comparative analysis of continuous variables across groups was conducted using the independent samples t-test for normally distributed data, and the Mann–Whitney *U* test for data not following a normal distribution. The chi-squared test was utilized for the assessment of categorical variables. To ascertain the predictors influencing the occurrence of a pseudoaneurysm requiring intervention, logistic regression was implemented**.** Statistical significance was established at a *p*-value of < 0.05. All statistical analyses were performed using SPSS software (version 29).

**Fig. 1:**
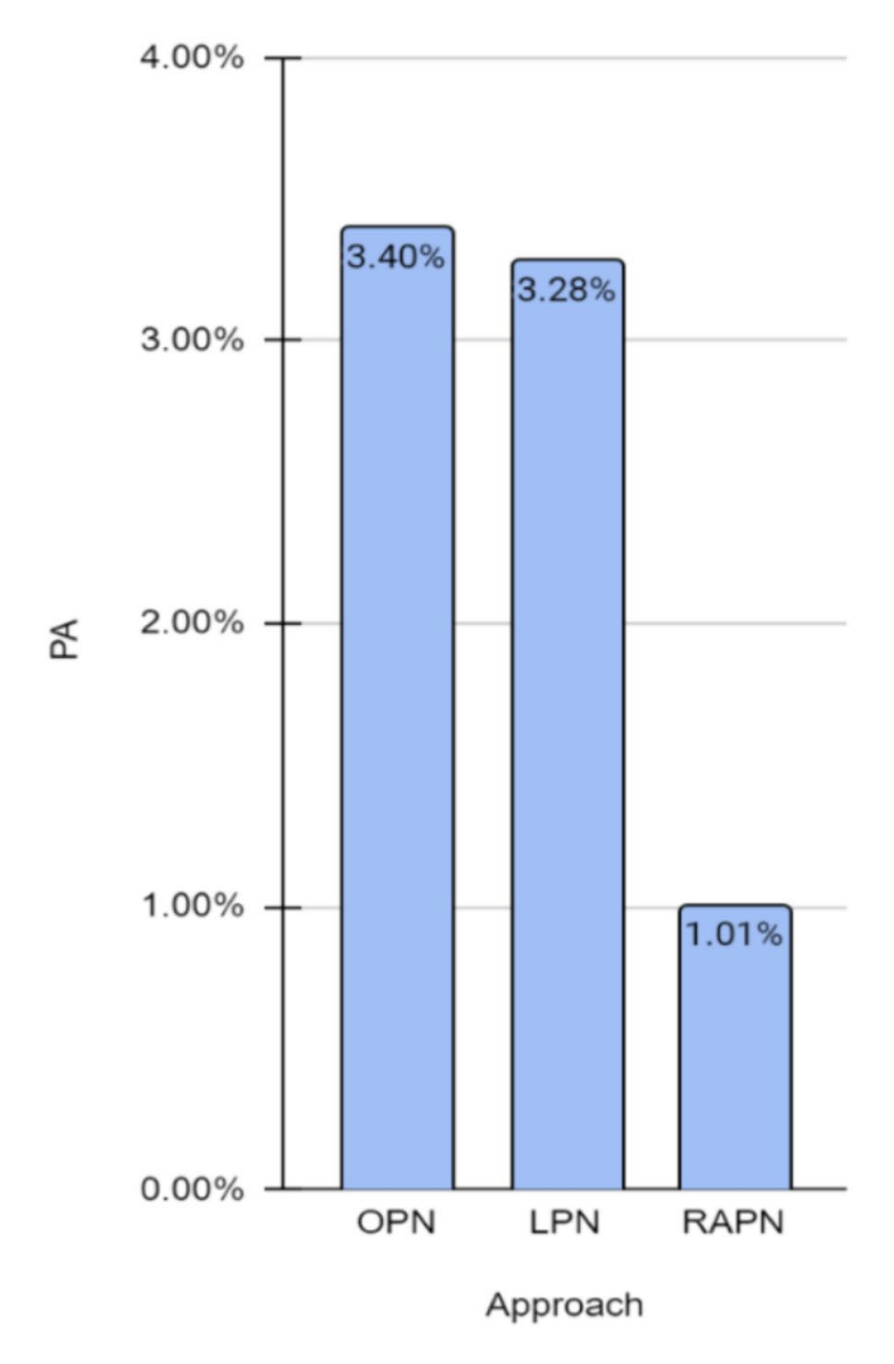
Incidence of RAP across surgical approach

## Results

A total of 785 patients underwent PN at our institution between January 2012 and December 2022. The surgical approaches included OPN (265 patients, 33.8%), LPN (122 patients, 15.5%), and RAPN (398 patients, 50.7%) (Fig. 1). A descriptive summary of demographics, preoperative tumor and surgical characteristics, and postoperative characteristics according to surgical methods are detailed in Table 1. The median age of our cohort was 63.9 years [IQR: 25–81 years], and 361 (46%) patients were females. Most of the patients were clinical T1a (*n* = 709, 90.2%) with a median tumor size of 3.5 cm according to preoperative imaging [IQR: 2.3–4.9] cm). RENAL nephrometry scores of 4–6, 7–9, and 10–12 for the entire cohort were 25.8%, 64.4%, and 9.7%, respectively. There was no difference between the RENAL nephrometry scores relative to the surgical methods (*p* = 0.73).

**Table 1:** Patient and tumor characteristics, surgical outcomes, and functional follow-up data

Variables	Pseudoaneurysm Present (*n* = 17)	Pseudoaneurysm Absent (*n* = 768)	*p-value*
Age, years, (± SD)	59.6 (12.2)	62.3 (6.2)	0.31
Male, *n* (%)	7 (47)	403 (44.2)	0.89
BMI, med (IQR)	25.6(22.9–29.9)	24.9(22.2–30.1)	0.43
ASA, med (IQR)	3 (2–3)	3 (1–4)	0.54
Preop eGFR, med (IQR)	76.2 (55.2–88.5)	80.3 (63.8–96.5)	0.21
Preop CKD, *n* (%)	4 (23.5)	154 (20)	0.54
Tumor size on CT, cm, med (IQR)	3.5 (1.6–4.7)	3.5 (2.3–4.9)	0.56
RENAL nephrometry score, n (%)			0.73
4–6 Low complexity	3 (17.6%)	140 (26.07%)	
7–9 Intermediate complexity	12 (70.5%)	345 (64.2%)	
10–12 High complexity	2 (11.7%)	52 (9.6%)	
Approach			0.04
RPN, *n* (%)	4 (1)	394 (0.98)	
LAP, *n* (%)	4 (3.3)	118 (0.96)	
OPN, *n* (%)	9 (3.4)	256 (0.96)	
Operation time, min, mean (± SD)	235.2 (75.5)	198.3 (71.5)	0.04
Ischemia time, min, median (IQR)	21.5 (12–32)	22.3 (17–28)	0.23
EBL, ml, med (IQR)	250 (150–350)	200 (100–300)	0.27
Time to embolization from surgery, days, med (IQR)	12 (1–21)	NA	
Last follow-up %eGFR preservation, median (IQR)	84.3 (69.7–97.2)	85.2 (76.3–97.8)	0.53

*ASA* American society of anesthesiologists, *BMI* body mass index, *CKD* chronic kidney disease, *CT* computed tomography, *eGFR* estimated glomerular filtration rate, *IQR* interquartile range, SD standard deviation

RAPs were diagnosed in 17 (2.1%) patients at a median of 12 days (range 1–31) since surgery. Table 2 details the patients’ and the tumors’ characteristics and the clinical presentation of all the pseudoaneurysm cases that required embolization. The presenting symptoms included massive hematuria (100%), flank pain (17%), clot urinary retention (23%), and hemorrhagic shock (5.8%). A single selective embolization of the bleeding vessel resulted in immediate cessation of bleeding in 16 of those patients (94%), while a second embolization was necessary in 1 patient (5.8%) (Table 3). No patient required or underwent surgery or nephrectomy to stop the bleeding. Only 3 (17.6%) patients were treated by blood transfusion, 2 of them by 1 unit of blood and 1 by 2 units. The time from the presentation to the emergency room to undergoing angiography among the entire cohort was 13.5 h. An analysis of the kidney function revealed no difference in the median percentage of the estimated glomerular filtration rate (eGFR) between patients who underwent embolization and those who did not (84.3 [69.7–97.2] versus 85.2 [76.3–97.8], *p* = 0.53, respectively). There were no intra- or post-procedure complications in association with RAP during angiography and embolization, such as renal artery dissection, groin hematoma, or bleeding. The preoperative platelet counts and coagulation test results were within the normal range among all patients who developed a postoperative RAP.

Table 3 summarizes the surgical variables. Patients who developed a postoperative RAP had a similar median intraoperative blood loss (250 [150–350] ml versus 200 [150–300] mL, *p* = 0.27), and a similar ischemia time (21.5 [12–32] min versus 22.3 [17–28] min, *p* = 0.23), compared to those who did not. The mean operative time for the patients with postoperative RAP was significantly longer than those without (235.2 [75.5] min versus 189.3 [71.5] min, respectively, *p* = 0.04). The presence of RAP was significantly more common following OPN and LPN compared to RAPN: 4 (3.3%), 9 (3.4%), and 4 (1%), respectively (*p* = 0.04). Age, sex, tumor side and size, RENAL nephrometry score, warm ischemia time, estimated blood loss, and clinical stage were comparable for the three surgical approaches (*p* > 0.05). Multivariable analyses confirmed that the surgical approach and operative time were the only significant predictors of the occurrence of a postoperative RAP (*p* < 0.05). There was no significant difference between patients with and those without RAP in terms of age, body mass index, ASA score, eGFR rate, number of patients with chronic kidney disease, preoperative clinical stage, tumor size, and RENAL nephrometry score (*p* > 0.05 for all).

**Table 2:** Patient and tumor characteristics and clinical presentation

Patient no.	Sex	Age, yrs	Renal Nephrometry Score					Renal Score	Tumor size, cm	Procedure	Ischemia type	Tumor pathology	Clinical presentation and postoperative days
			R	E	N	A	L						
1	F	62	1	2	1	A	2	6	3	Robotic	Warm	Clear cell	Abdominal pain, gross hematuria
2	M	59	2	3	1	A	2	8	4.5	Robotic	Warm	Papillary	Hemorrhagic shock, gross hematuria
3	M	60	1	2	3	A	2	8	3.2	Robotic	Warm	Clear cell	Gross hematuria
4	F	60	1	2	2	A	3	8	1.6	Robotic	Warm	Papillary	Gross hematuria
5	M	64	1	2	1	A	3	7	2.3	Laparoscopic	Warm	Clear cell	Urinary retention, gross hematuria
6	M	52	2	1	3	A	2	8	4.7	Laparoscopic	Warm	Clear cell	Gross hematuria
7	M	54	1	2	1	A	3	7	2.3	Laparoscopic	Warm	Clear cell	Urinary retention, gross hematuria
8	F	76	1	3	3	P	1	8	3.1	Laparoscopic	Warm	Papillary	Flank pain, gross hematuria
9	M	60	1	2	2	X	2	7	3	Open	Warm	Clear cell	Hemorrhagic shock
10	M	60	3	2	3	X	3	11	7	Open	Warm	Papillary	Gross hematuria, flank pain
11	M	52	2	2	3	A	3	10	4.3	Open	Warm	Clear cell	clot retention, gross hematuria
12	M	62	2	2	3	P	3	10	6.5	Open	Warm	Clear cell	Gross hematuria, clot retention
13	F	60	1	3	3	A	3	10	3.5	Open	Warm	Clear cell	Abdominal pain, gross hematuria
14	M	57	2	2	2	P	3	9	5.2	Open	Warm	Clear cell	Gross hematuria
15	M	52	1	3	1	A	1	6	1.4	Open	Warm	Chromophobe	Gross hematuria, Flank pain
16	M	58	2	1	1	P	2	6	4.2	Open	Warm	Clear cell	Gross hematuria
17	M	65	2	2	2	A	3	9	4.3	Open	Warm	Papillary	Gross hematuria

**Table 3:** Clinical and surgical parameters across nephrectomy approaches

	Total (785)	Open partial nephrectomy (*n* = 265)	Laparoscopic partial nephrectomy (*n* = 122)	Robotic-assisted partial nephrectomy (*n* = 398)	*p* value
Age, yrs. (± SD)	62.2 (6.3)	64.3 (4.1)	62.3 (8.1)	59.2 (10.2)	0.31
Male, n (%)	401 (51%)	125 (47.1%)	59 (48.3%)	206 (51.7%)	0.71
Side, Rt (%)	409 (52.1%)	119 (44.9%)	63 (51.63%)	200 (50.25%)	0.83
Tumor size cm, med (IQR)	3.53 (2.4–4.7)	3.51 (2.6–5)	3.49 (2.7–4.8)	3.51 (2.2–4.9)	0.53
RENAL nephrometry score	7 (5–9)	8 (5–9)	7 (6–9)	7 (5–9)	0.55
Warm ischemia time (min)	22.3 [12–41]	24.3 [12–32]	23.4 [9–33]	22.4[12–32]	0.45
Estimated blood loss (ml)	200 [50–400]	230 [50–400]	255 [50–350]	180 [50–350]	0.55
ASA	3 (2–4)	3 (2–4)	3 (2–4)	3 (2–4)	0.54
Clinical stage					0.67
CS T1a	709 (90.2%)	240 (90.56%)	107 (87.7%)	362 (90.95%)	
CS T1b	76 (9.8%)	25 (9.4%)	15 (12.29%)	36 (9.1%)	
Benign pathology	14% (95% CI: 0.13–0.16)	15% (95% CI: 0.13–0.17)	18% (95% CI: 0.16–0.22)	13% (95% CI: 0.12–0.15)	0.56
Post-operative pseudoaneurysm	17 (2.1%)	9 (3.4%)	4 (3.3%)	4 (1%)	0.04
Time to embolization, days (range)	12 (1–31)	13 (1–20)	11 (7–18)	16 (5–31)	0.63

## Discussion

Our study showed a significantly decreased incidence of RAPs following RAPN compared to LPN and OPN approaches in a tertiary cancer center during the last decade. Specifically, the incidence of RAPs was 1%, 3.3%, and 3.4%, respectively. In addition to surgical type, the only other significant variable for the development of RAP was the length of operative time (*p* < 0.004), while all of the other evaluated variables, including demographics (gender, age) and surgical characteristics (tumor size, side, and RENAL nephrometry score) were not. The relative risk of developing a RAP following RAPN was 0.29–0.3 compared to LPN and OPN. To the best of our knowledge, this study represents the first documentation supporting the advantage of RAPN in terms of the incidence of RAPs among patients treated by PN for renal tumors.

Hemorrhage is the most frequent serious complication of PN, either intraoperatively or during the postoperative period. Delayed hemorrhage is an infrequent event, with an incidence of 0.4–5% [2, 3, 7, 8] and it usually due to RAP. We believe that the development of RAP is probably related to an injured branch of the renal artery during the tumor resection or to the renorrhaphy following the tumor resection leading to blood extravasation to the collecting system or the perinephric space. Possible suggested risk factors of RAPs after PN including the surgical approach for the PN, technical surgical modifications, and tumoral complexity [11–15].

Since the introduction and wide acceptance of OPN as an effective and safe surgical option for a localized renal mass, MIPN (such as LPN and RAPN) was developed to decrease to decrease the rates of adverse reactions of surgery and hospitalization length [6–9]. The current findings showed, for the first time for a decreased rate of RAP among patients who undergo RAPN compared to the open and laparoscopic approaches. Ghoneim et al [12] described 1461 patients who underwent PN among whom 15 developed RAP post-PN: 0.6% post-OPN and 2.6% post-LPN. Contrarily, no difference in the risk of RAP was found by Chavali [13] who compared RAPN to OPN, or by Saoud [14] who compared MIPN to OPN. Jain et al [15] performed a systematic review of the incidence and risk factors of RAPs following PN in a multicenter cohort of 5229 patients, among whom 77 (1.47%) developed RAPs. Those authors [15] reported that the incidence of RAPs after an open approach was 1% compared to 1.96% after MIPN. They offered a possible explanation that the higher incidence of RAP after MIPN resulting of the higher rate of the use of renorrhaphy during the procedure. Renorrhaphy can lead to arteriole laceration which later creates RAPs following the closure of the renal defect [15]. The major limitations of Jain et al.’s meta-analysis included the lack of information regarding the tumor complexity and therefore a significant patient bias could not be ruled out. Second, their MIPNs included mainly LPNs. Leow et al.’s [16] meta-analysis compared RAPN with LPN outcomes of 4919 patients (54% RAPN). The patients who had been treated with RAPN had a larger tumor size and higher RENAL nephrometry scores. Despite that all-complication and major complication rates were significantly lower in the RALP group compared to the LPN group. Hyams et al.’s [17] multi-institute report on 998 patients after MIPN (including LPN and RAPN) cited an incidence of iatrogenic vascular lesions of 2%, similar to our rate of 2.1%. Most of their patients (85%) had RAPs while the rest had arteriovenous fistulas [17]. That report did not include any data on the percentage of patients who underwent LPN compared to RAPN or on any differences in the complication rate in general and specifically for the incidence of post-operative RAP [17].

Chavali et al.’s [13] estimations for the higher incidence of RAP after MIPN included the option of a less precise suturing of laparoscopic technique. In contrast, the robotic approach has several advantages, including three-dimensional visualization, improved visual magnification, and mechanical dexterity that translate to better surgical outcomes. The findings of several retrospective analyses that compared the surgical and functional outcomes of RAPN with those of LPN favored robotic surgery by its affording shorter ward ischemia time [2–4], better postoperative renal function [2–4], shorter operative time [3], and shorter hospital length of stay [4]. Our earlier results support these differences [17]. To the best of our knowledge, the current study is the first to demonstrate a significantly reduced rate of RAPs following a robotic approach compared to open and laparoscopic approaches.

The presence of RAP is associated with a significant impact on the recovery process following surgery due to a need for an urgent invasive therapeutic procedure for selective arterial embolization (SAE), possible hemodynamic instability, and frequent use of blood transfusion [18].

Operative time was the other variable associated with the risk for RAP (both univariate and multivariate analysis, *p* < 0.05). One possible explanation for this observation is that operative time could serve as a surrogate marker for the complexity of the tumor. We did not, however, establish any correlations between ischemia time, estimated blood loss, RENAL nephrometry score, tumor size, and the risk of RAP. Chavali [13] also did not find any correlation between the RENAL nephrometry score and the risk for RAP development, while Saoud [14] et al. demonstrated that a higher RENAL nephrometry score was associated with the increased probability of RAP after PN, as did Nadu et al [23] in a series of LPNs [13]. The systematic review by Jain [15] was not able to correlate tumor complexity and the risk of RAP, calling for further investigations on these issues.

 Study by Giuliano [5] randomized 208 patients to LPN versus OPN, and those authors demonstrated decreased abdominal wall complications and better renal function at 3 and 12 months from surgery in favor of LPN [5].

A systemic review for RAPN versus OPN demonstrated a lower rate of post-operative complications for RAPN, but the analysis was used for all complication rates and thus no data are available for RAP specifically.

The embolization of RAPs was safe, effective, and not associated with any complications in our cohort. Sixteen (92%) of our 17 patients experienced immediate cessation of bleeding after they underwent an SAE for symptomatic RAPs after PN, while the additional patient needed an additional SAE procedure to stop the bleeding. None of those patients were treated surgically for RAPs. Notably, the eGFR was maintained in the patients who underwent angiography and selective embolization comparable to that of the group of patients who did not require any endovascular intervention (84.3 versus 85.2 mL/min, *p* = 0.53). SAE for controlling renal biopsy-related arteriovenous fistulas was first reported in 1973 [17, 18]. Since then, it has become the first-line therapy for iatrogenic vascular lesions after urologic procedures, such as PN and percutaneous nephrolithotomy, and several studies have demonstrated the safety and efficacy of SAE for controlling hemorrhagic complications following PN [17–19]. The efficacy of SAE for RAP in our cohort aligns closely with those reported in currently available studies, emphasizing the effectiveness of this approach [17–19].

None of our patients underwent a surgical procedure or nephrectomy due to RAP. In addition to its safety, another benefit of SAE is the lack of effect on immediate and prolonged renal function [16–19]. Several papers have demonstrated the lack of any deterioration of renal functioning due to RAP [18–20] however, only two of them had prolonged the follow-up period beyond the time of the procedure [18–20].

A RAP can be diagnosed by contrast-enhanced imaging, usually computerized tomographic (CT) scanning or angiography for patients presenting with clinical suspicion for RAP [16–18]. There is currently no flow chart to guide the initial imaging of choice for a given patient presenting with late bleeding following PN. The use of an immediate CT scan can potentially eliminate the need for invasive procedures, such as SAE [16–18], that RAP has not been identified. However, a CT scan use can prolong the time to SAE and an additional dose of contrast material and radiation will be used in case the imaging shows the presence of RAP [17–19]. Determination of the imaging of choice in cases of RAP following PN was beyond the scope of the current study: all our patients underwent angiography followed by embolization.

An additional clue for the presence of a RAP after PN is the timing of its development from the time of surgery [19, 20]. The median time from surgery to the presentation of RAP in our study was 12 days (range 1–31), similar to the findings of others. Nevertheless, there are several reports demonstrating of the first clinical evidence of RAP up to 6 months following surgery, warranting longer term clinical suspicion.

Only three of our patients (17.6%) were treated by blood transfusions. That low incidence derives from several factors. The time from presentation to angiography in the present cohort was 13.5 h, and only two (11.7%) of the cohort presented with hemodynamic instability, which alerted to the possibility that the extent of bleeding would not likely be severe. However, since we are unable to determine the extent of bleeding and the potential for bleeding progression tendency at the time of presentation, immediate steps should be taken when a RAP is suspected for the diagnosis and treatment of the bleeding source.

Several limitations to our study are mentioned. First, its retrospective design inherently introduces potential sources of bias despite the use of several basic demographics, tumor size, and complexity that could serve to control for potential bias. Although our study encompassed a sizable population of 785 patients, the infrequent occurrence of pseudoaneurysms posed a challenge in discerning factors that could be associated independently with the development of RAPs**.**

## Conclusions

RAPN is an uncommon but potentially life-threatening complication following PN. RAPN is associated with a significantly decreased rate of postoperative pseudoaneurysm compared to OPN and LPN. High index of suspicion and understanding of its typical clinical presentation will enable expeditious diagnosis. Selective embolization is an efficient and safe treatment for RAPs following PN.

## Data Availability

No datasets were generated or analysed during the current study.
